# Enterococcus-driven metabolite-host gene networks in IBD-associated colorectal carcinogenesis: integrative multi-omics and experimental validation

**DOI:** 10.3389/fcell.2026.1793350

**Published:** 2026-03-30

**Authors:** Gang Huang, Jiani Liu, Zhipeng Cheng, Yixin Chen, Kexin Chen, Wan Liu, Hancong Liu, Xinhao Xia, Man Lu, Wenxi Cui, Qingqing Zhang, Yi Yuan, Fei Zhong, Yiwen Liao

**Affiliations:** 1 Department of Laboratory Medicine, Ganzhou Cancer Hospital, Ganzhou, Jiangxi, China; 2 Department of Traditional Chinese Internal Medicine, School of Traditional Chinese Medicine, Southern Medical University, Guangzhou, China; 3 Department of Anesthesiology, Shanghai Pulmonary Hospital, School of Medicine, Tongji University, Shanghai, China; 4 The First School of Clinical Medicine, Southern Medical University, Guangzhou, China; 5 The School of Public Health, Guangzhou Medical University, Guangzhou, China; 6 The School of Integrated Traditional Chinese and Western Medicine, Guangzhou Medical University, Guangzhou, China; 7 Department of Rheumatology and Clinical Immunology, The First Affiliated Hospital of Guangzhou Medical University, GuangZhou, China; 8 Department of Rheumatology and Immunology, Nanfang Hospital, Southern Medical University, Guangzhou, Guangdong, China; 9 Department of Laboratory Medicine, Guangdong Provincial People’s Hospital, Guangdong Academy of Medical Sciences, Southern Medical University, Guangzhou, China

**Keywords:** bioinformatics, colitis, colorectal cancer, enteritis, *Enterococcus*

## Abstract

**Background:**

This study aims to elucidate the role of Enterococcusin the progression from inflammatory bowel disease to colorectal cancer (CRC), with a focus on identifying key metabolites and host genes regulated by Enterococcusand their influence on CRC development.

**Methods:**

Using the database gutMGene, gutMDisorder and MACdb, we mined the key metabolites and human genes. We acquired the activated genes (panel 1) and inhibited genes (panel 2), and metabolite associated genes (MAGs, panel 3). Subsequent analyses included protein-protein interaction (PPI) network construction, functional enrichment, differential expression and survival analysis in CRC, and immune infiltration assessment. *In vitro* experiments validated the regulatory effects of *E. faecalisand* its key metabolites on candidate genes. Single-cell RNA sequencing (scRNA-seq) was used to dissect cell-type-specific expression patterns within the tumor microenvironment.

**Results:**

We screened 12 activated genes (Panel1: *IL11, IL24, IFNG, IL10, IL12B, IL1B, IL6, TNF, ANGPTL4, CXCL10, PLIN2,* and *PPARG*) and four inhibited genes (Panel 2: *CXCL8, IL6, TNF,* and *PDCD6IP*). Three metabolites were found important in CRC development: agmatine, formate, and levodopa, linking with 28 MAGs. In particular, *IL10, IL11, CXCL10, IL1B,* and *IFNG* are protective genes in CRC; and there are four MAGs associated with CRC PFS, and they are all survival-risk genes: *COMT, PRL, EDNRA,* and *MAPK3*. Experimental validation showed that *E. faecalis* significantly upregulated the level of *IL-10* and *IL-1B* in CRC, while its metabolites agmatine and levodopa markedly induced the expression of the survival-risk gene *MAPK3*. scRNA-seq revealed cell-type-specific expression patterns, where *IL1B* was significantly upregulated in both tumor epithelial and myeloid cells, and *IL10* was specifically elevated in tumor epithelial cells. In contrast, *MAPK3* exhibited divergent trends, showing downregulation in tumor epithelial cells but significant upregulation in myeloid cells.

**Conclusion:**

*Enterococcus* exhibits a dual role in colitis-associated CRC, correlating with both tumor-suppressing and tumor-promoting effects. It may activate protective immune genes while its metabolites, agmatine and levodopa, alter the expression of oncogenic MAGs. The findings highlight the complex metabolite-host gene networks driven by Enterococcusand suggest estradiol and sodium arsenite as potential adjuvant therapies, offering new insights into precision intervention for CRC.

## Introduction

Chronic inflammation of the intestine is strongly associated with the development of colorectal cancer (CRC). In this progress, chronic inflammation-driven carcinogenesis is the most well-known pathogenic factor. Long-term intestinal inflammation leads to the sustained activation of immune cells (such as macrophages and neutrophils), which release large amounts of reactive oxygen species or reactive nitrogen species, and pro-inflammatory factors. These substances directly damage the DNA of intestinal epithelial cells, inducing gene mutations. It is generally believed that chronic inflammation leads to DNA damage induced by oxidative stress, thereby activating oncogenes and inactivating tumor suppressor genes (Hnatyszyn et al., 2019). In the sequence of inflammation-dysplasia-carcinoma, oxidative damage and DNA double-strand break gradually increase. Inflammation and carcinogenic processes are driven by the host’s immune response and intestinal microbiota (and their metabolic products) (Quaglio et al., 2022; Shah and Itzkowitz, 2022). Therefore, in-depth research into the role of intestinal microbiota and their interaction with CRC is crucial for exploring the pathogenesis of CRC and developing treatment strategies. The inflammatory bowel diseases (IBD), ulcerative colitis (UC) and Crohn’s disease (CD) are chronic inflammatory disorders associated with CRC. However, due to the long total length of the intestines, the complex microenvironment, and the extremely lengthy process of carcinogenesis, there remains much uncertainty regarding the pathogenesis of enteritis/colitis-associated CRC.

Several important effects of the gut microbiota are as follows. First, patients with enteritis/colitis often exhibit dysbiosis, especially with reduction in beneficial bacteria and an increase in potentially pathogenic bacteria (such as *Fusobacterium* nucleatum and enterotoxin-producing *Escherichia coli*). Some pathogenic bacteria secrete gene toxins (colibactin), which directly induce DNA double-strand breaks and accelerate gene mutations. Second, the metabolic products can play a significant role in carcinogenesis. For example, harmful bacteria metabolize to produce secondary bile acids (such as deoxycholic acid) and hydrogen sulfide, which damage epithelial cells, promote oxidative stress, and activate carcinogenic pathways; meanwhile, the reduction in short-chain fatty acids (such as butyrate) produced by beneficial bacteria weakens their protective effects, including anti-inflammatory properties, maintenance of barrier integrity, and induction of cancer cell apoptosis. Therefore, there have been studies suggesting that gut microbiota transplantation can be used as an adjunctive prevention against or treatment for CRC (Song et al., 2024). Additionally, microbiota-immune interactions may lead to immune dysregulation in the intestinal epithelial microenvironment, promoting tumorigenesis and immune escape. Disrupted microbiota structure damages the mucosal barrier, increases pathogen translocation, and continuously stimulates the immune system, forming a vicious cycle of inflammation/microbiota dysbiosis/carcinogenesis. Therefore, gut microbial and their metabolites participate largely in the mechanisms of CRC development. Among them, *enterococcus* is one of the most common intestinal flora members. *Enterococcus* species is Gram + bacteria in the gut microbiota. Due to the dual nature, strains of this species have sparked considerable debate (Archambaud et al., 2024); they can either be utilized as probiotics in the food industry or demonstrate resistance to antibiotics, potentially leading to severe illness, even death (Boeder et al., 2024). *Enterococcus* can induce endocarditis (Sunbul et al., 2018), gastritis (El-Zimaity et al., 2003), apical periodontitis (Ma et al., 2024), etc. Besides, it can secrete emergent *enterococcus* toxins, leading to multidrug resistant infections (Xiong et al., 2022; York, 2022). In summary, the effects of *enterococcus* on the host are extraordinarily complex. Currently, there is still extremely limited knowledge regarding the role *enterococcus* plays in the progression of inflammatory intestine diseases to CRC. This study aims to clarify this role, in particular, we sought to identify key metabolites and human genes regulated by *enterococcus* and elucidate how they influence the development of CRC.

## Materials and methods

### Metabolites and human genes affected by *Enterococcus*

Using the database gutMGene and gutMDisorder, we confirmed the role of *Enterococcus* and its change direction in intestine diseases and colorectal cancer (CRC), as well as the human genes and metabolites directly affected by *Enterococcus*. We acquired the activated genes (panel 1) and inhibited genes (panel 2), and the typical metabolites from *Enterococcus*. Furthermore, in order to focus on the key molecular mechanisms in the progression from colitis to colorectal cancer, we utilized the MACdb database to screen for metabolites that were consistently upregulated in CRC (as the key metabolites). Next, we used the Zeroz Crosslinks data of the PubChem database to search for genes associated with the key metabolites. PubChem is an open chemistry database at the National Institutes of Health (NIH). It provides information about chemical structures, identifiers, chemical and physical properties, biological activities, patents, health, safety, toxicity data, etc. All the metabolite associated genes (MAGs, as panel 3) were also used to analyze the mechanism underlying enteritis/colitis-CRC progression. In order to compile evidence on Enterococcus-related diseases, bar graphs were drawn by the Yangbo studio online visualization tool (http://yangbostudio.cn).

### Protein-protein interaction (PPI) and enrichment analysis

Protein-protein interaction (PPI) networks of activated genes (Panel 1), inhibited genes (Panel 2), and metabolite-associated genes (Panel 3) were constructed using the STRING database (version 11.5, https://string-db.org/). Gene symbols were uploaded with organism restricted to *Homo sapiens*. Interaction sources included experiments, curated databases, co-expression, gene neighborhood, gene fusion, and co-occurrence under default settings. The minimum required interaction score was set at 0.4 (medium confidence). The generated interaction networks were exported and visualized using Cytoscape (version 3.9.x), and hub genes were identified based on degree centrality calculated by the NetworkAnalyzer plugin. Functional enrichment analysis was simultaneously performed using the STRING enrichment module, including Gene Ontology (GO: Biological Process, Cellular Component, Molecular Function), KEGG pathways, Reactome pathways, WikiPathways, and subcellular localization (COMPARTMENTS). P values were adjusted using the Benjamini–Hochberg method, and terms with false discovery rate (FDR) < 0.05 and gene count ≥3 were considered significantly enriched. The top enriched terms were ranked according to adjusted p values and visualized using R (version 4.2.x) with the ggplot2 package.

### Differential expression and survival analysis in CRC

Differential gene expression analysis in colorectal cancer was conducted using the GEPIA2 online platform (http://gepia2.cancer-pku.cn/), which integrates RNA sequencing data from TCGA and GTEx. Gene expression levels were analyzed in the TCGA COAD and READ datasets using log2 (TPM + 1) normalization. Tumor tissues were compared with normal tissues under default GEPIA2 parameters, and genes with |log2 fold change| ≥ 1 and q value <0.01 were considered differentially expressed genes (DEGs). For survival analysis, progression-free survival (PFS) in combined TCGA COAD and READ cohorts was evaluated using the GEPIA2 survival module. Patients were divided into high- and low-expression groups based on median gene expression levels. Kaplan–Meier survival curves were generated and compared using the log-rank test. Genes with log-rank p < 0.05 were considered significantly associated with PFS, and hazard ratios (HRs) were used to classify genes as survival-risk genes (HR > 1) or protective genes (HR < 1).

### Immune infiltration analysis

The correlation between key genes and tumor immune infiltration was evaluated using the TIMER2.0 database (http://timer.comp-genomics.org/). Partial Spearman correlation analysis adjusted for tumor purity was performed to assess associations between gene expression and infiltration levels of B cells, CD8^+^ T cells, CD4^+^ T cells, macrophages, neutrophils, and dendritic cells in TCGA COAD and READ samples. Correlation coefficients (rho values) and corresponding p values were extracted, and p < 0.05 was considered statistically significant. These analyses were used to infer potential immune regulatory mechanisms underlying Enterococcus-driven gene networks in CRC.

### RT-qPCR analysis

Total RNA was extracted from treated colorectal cancer cells using TRIzol reagent according to the manufacturer’s instructions. RNA purity and concentration were measured using a NanoDrop spectrophotometer. One microgram of total RNA was reverse-transcribed into cDNA using a reverse transcription kit following standard protocols. Quantitative PCR was performed using SYBR Green Master Mix on a real-time PCR detection system under the following cycling conditions: initial denaturation at 95 °C for 3 min, followed by 40 cycles of 95 °C for 10 s and 60 °C for 30 s. Melting curve analysis was performed after amplification to verify specificity. PCR products were further examined by 1.5% agarose gel electrophoresis to confirm single bands of expected size, and representative amplicons were validated by Sanger sequencing to ensure sequence accuracy. Relative gene expression levels were calculated using the 2^−ΔΔCt method with GAPDH as the internal reference gene. All experiments were performed with three independent biological replicates and technical triplicates. Primer sequences (5′-3′) were as follows:

**Table:** 

Gene	Forward	Reverse
MAPK3	GCT​AAT​GAC​TAG​GAG​GTG​ACT​GAG​G	ACA​GAA​TAG​GCA​ACA​AGG​CAA​GAA​C
IL-1β	CCG​ACC​ACC​ACT​ACA​GCA​AGG	GGG​CAG​GGA​ACC​AGC​ATC​TTC
IL-10	AGC​AGC​CAG​AGG​GTT​TAC​AAA​GG	CAG​GAG​CCA​AAG​GTG​AGT​GAG​AG

### Single-cell RNA sequencing (scRNA-seq) analysis

Single-cell RNA sequencing data (GSE132465) were analyzed using the Seurat package (version 4.3.x) in R (version 4.2.x). Cells with fewer than 200 detected genes or mitochondrial gene content exceeding 10% were excluded to ensure data quality. Data were normalized using the LogNormalize method with a scale factor of 10,000, and 2,000 highly variable genes were identified using the “vst” method. Principal component analysis (PCA) was performed, and significant principal components were selected based on elbow plot inspection. Cells were clustered using the Louvain algorithm and visualized using UMAP. Cell types were annotated according to canonical marker genes for epithelial and myeloid populations. Differential expression analysis between tumor and normal samples within specific cell types was performed using the Wilcoxon rank-sum test, and adjusted p < 0.05 was considered statistically significant.

### Differentially expressed genes in CRC among key-gene panels

For three panels (activated genes, inhibited genes, and MAGs), we analyzed each gene individually intending to identify which genes have altered expression in CRC. The gepia2 tool was used to compare tumor samples and normal samples in the TCGA CRC datasets (COAD and READ), and the differentially expressed genes (DEGs) were shown as box plots.

### Links between CRC progression free survival and key genes

Again, for three panels (activated genes, inhibited genes, and MAGs), we used the gepia2 tool was used to analyze the links between the expression of each gene and CRC progression free survival (PFS) in the TCGA CRC datasets (COAD and READ). For genes with a Log-rank p value <0.05, it was divided into the survival-risk gene or protective gene according to the HR value. And the survival curve was plotted for each survival-risk or protective gene.

### The influence of key genes on the tumor immune microenvironment

We mined the potential influence of key genes on the tumor immune microenvironment using the Tumor IMmune Estimation Resource (TIMER) database, which is a comprehensive resource for systematical analysis of immune infiltrates across diverse cancer types. For CRC DEGs, CRC survival-risk and CRC protective genes, immune infiltration was analyzed, regarding Purity, B Cells, CD8^+^ T Cells, CD4^+^ T Cells, Macrophages, Neutrophils, and Dendritic Cells, in the TCGA COAD and READ samples. Through immune infiltration analysis, we aimed to speculate on the immune cell mechanisms by which key metabolites and genes potentially influence CRC development.

### Potential drug screening

Potential inhibitory compounds targeting CRC survival-risk genes were identified using the Comparative Toxicogenomics Database (CTD, http://ctdbase.org/). For each survival-risk gene, chemicals reported to decrease gene expression, abundance, activity, or accumulation were retrieved, and only curated interactions with direct experimental evidence were included. After removing duplicate compounds, chemicals were ranked based on the number of survival-risk genes they simultaneously inhibited. Compounds targeting at least two survival-risk genes were prioritized and visualized using R software.

### Statistical analysis

All quantitative data are presented as mean ± standard deviation (SD). Statistical analyses were performed using GraphPad Prism (version nine.x) and R (version 4.2.x). Comparisons between two groups were conducted using two-tailed Student’s t-tests. Comparisons among multiple groups were performed using one-way ANOVA followed by Tukey’s *post hoc* test. Survival differences were evaluated using the log-rank test as implemented in GEPIA2. Correlation analyses were performed using Spearman’s rank correlation. A p value <0.05 was considered statistically significant.

## Results

### Key genes and metabolites associated with enteritis/colitis-CRC progression

**FIGURE 1:**
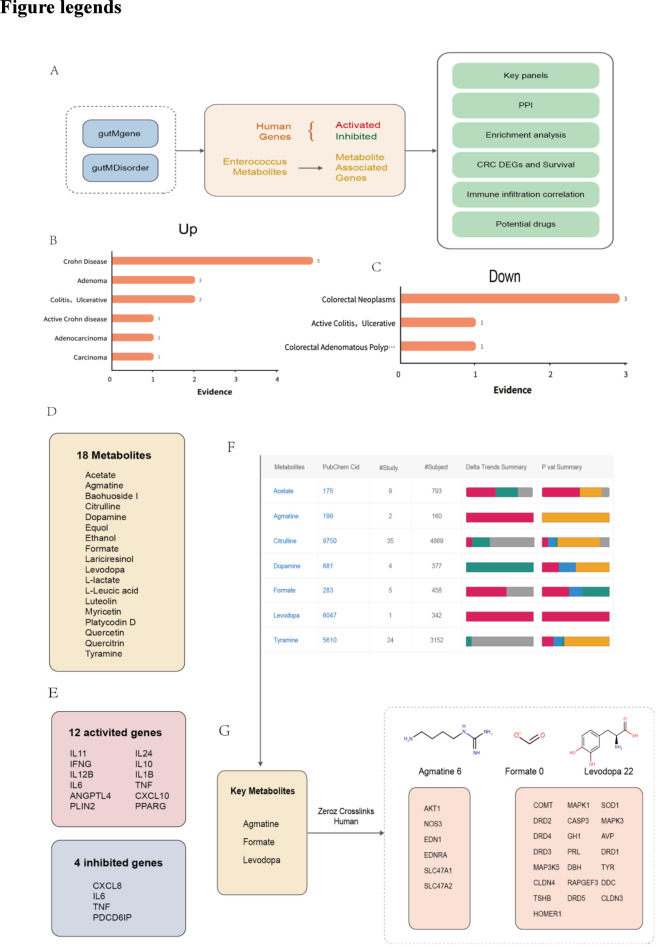
Workflow and Key Genes/Metabolites in Enteritis/Colitis-CRC Development **(A)** The analysis flow chart of this study **(B)** In the gutMDisorder database, the number of evidence supporting that *enterococcus* abundance increases in inflammatory intestine diseases and colorectal cancer. **(C)** In the gutMDisorder database, the number of evidence supporting that *enterococcus* abundance decreases in inflammatory intestine diseases and colorectal cancer. **(D)** There are 18 typical metabolites of *enterococcus* from the gutMGene database. **(E)** There are 12 activated genes (Panel1: IL11, IL24, IFNG, IL10, IL12B, IL1B, IL6, TNF, ANGPTL4, CXCL10, PLIN2, and PPARG) and four inhibited genes (Panel 2: CXCL8, IL6, TNF, and PDCD6IP) in the gutMGene database. IL6 and TNF are both activated genes and inhibited genes. **(F)** Key metabolites in the MACdb database, with their change trend in CRC (for directions, red: increased; green: decreased; for P values, red: p ≤ 0.001; blue: 0.001 < p ≤ 0.01; green: 0.01 < p ≤ 0.05; yellow: p > 0.05). After screening the metabolites consistently increased in CRC, following three metabolites are obtained: Agmatine, Formate, and Levodopa. **(G)** Then, the MAGs of these three metabolites are extracted from the Zeroz Crosslinks data of the PubChem database (Figure 3B). There were six gene targets of agmatine, and 22 gene targets of levodopa. No targets were found by formate. These 28 MAGs (AKT1, NOS3, EDN1, EDNRA, SLC47A1, SLC47A2, COMT, MAPK1, SOD1, DRD2, CASP3, MAPK3, DRD4, GH1, AVP, DRD3, PRL, DRD1, MAP3K5, DBH, TYR, CLDN4, RAPGEF3, DDC, TSHB, DRD5, CLDN3, HOMER1) were used as panel 3.

In the gutMDisorder database (Figures 1B,C), most evidence indicates that *enterococcus* abundance increases in inflammatory intestine diseases and colorectal cancer, which suggests that increased abundance of *enterococcus* is associated with enteritis/colitis-CRC progression, and its detection in the gut microbiota may serve as a potential marker for enteritis/colitis or CRC. However, the specific causal relationships remain unknown at present. Subsequently, we obtained 18 typical metabolites of *enterococcus* from the gutMGene database (Figure 1D). In addition, there were 12 activated genes (Panel1: *IL11, IL24, IFNG, IL10, IL12B, IL1B, IL6, TNF, ANGPTL4, CXCL10, PLIN2,* and *PPARG*) and four inhibited genes (Panel 2: *CXCL8, IL6, TNF,* and *PDCD6IP*) were found in the gutMGene database (Figure 1E). *IL6* and *TNF* are both activated genes and inhibited genes.

Next, we evaluated each metabolite in the MACdb database and screened the key metabolites (consistently increased in CRC, Figure 1F). Following three metabolites were obtained: Agmatine, Formate, and Levodopa. Then, the MAGs of these three metabolites were extracted from the Zeroz Crosslinks data of the PubChem database (Figure 1G). There were six gene targets of agmatine, and 22 gene targets of levodopa. No targets were found by formate. These 28 MAGs (*AKT1, NOS3, EDN1, EDNRA, SLC47A1, SLC47A2, COMT, MAPK1, SOD1, DRD2, CASP3, MAPK3, DRD4, GH1, AVP, DRD3, PRL, DRD1, MAP3K5, DBH, TYR, CLDN4, RAPGEF3, DDC, TSHB, DRD5, CLDN3, HOMER1*) were used as panel 3.

### PPI and enrichment analysis

**FIGURE 2:**
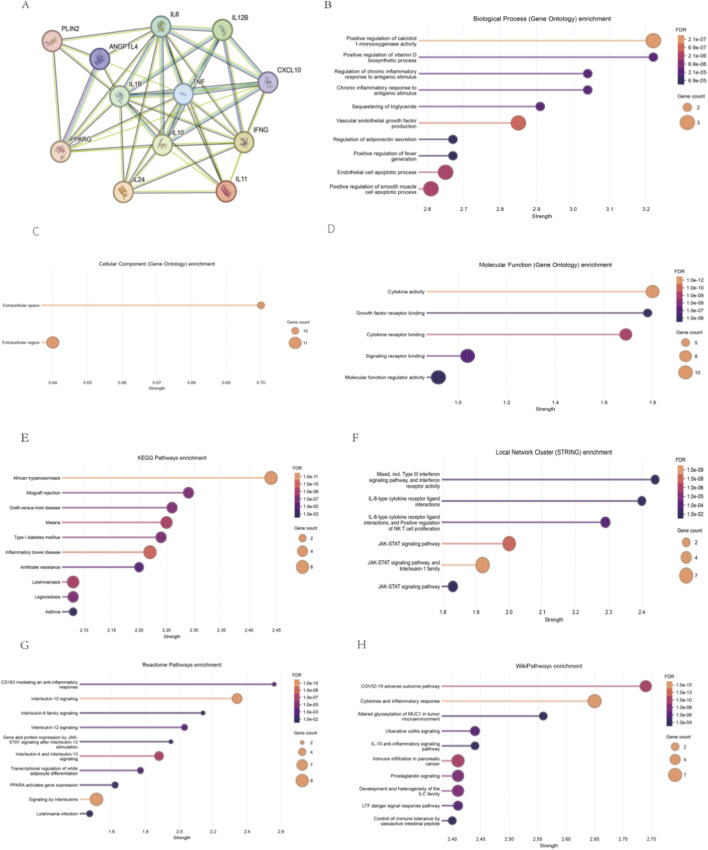
PPI and Enrichment analysis of Panel 1 **(A)** The PPI network of 12 activated genes in Panel 1. **(B)** The enriched GO BP terms based on genes in Panel 1. **(C)** The enriched GO CC terms based on genes in Panel 1. **(D)** The enriched GO MF terms based on genes in Panel 1. **(E)** The enriched KEGG pathways based on genes in Panel 1. **(F)** The enriched local network cluster in the STRING database based on genes in Panel 1. **(G)** The enriched reactome terms based on genes in Panel 1. **(H)** The enriched Wiki-pathways based on genes in Panel 1.

Based on panels 1, two and 3, the PPI networks were generated, and enrichment analysis was performed. For panel 1, *CXCL10, IFNG, IL10* and *IL1B* were hub nodes among the 12 activated genes (Figure 2A). The enriched GO terms were presented in Figures 2B–D, and the top enriched BP terms include Positive regulation of calcidiol 1-monooxygenase activity, Positive regulation of vitamin D biosynthetic process, Chronic inflammatory response to antigenic stimulus, Regulation of chronic inflammatory response to antigenic stimulus, Sequestering of triglyceride, Vascular endothelial growth factor production, Positive regulation of fever generation, Regulation of adiponectin secretion, Endothelial cell apoptotic process, and Positive regulation of smooth muscle cell apoptotic process. There were two enriched CC terms: Extracellular region, and Extracellular space, as well as five enriched MF terms: Cytokine activity, Cytokine receptor binding, Signaling receptor binding, Molecular function regulator activity, and Growth factor receptor binding. The top 10 enriched KEGG pathways included (Figure 2E): African trypanosomiasis, Allograft rejection, Graft-versus-host disease, Malaria, Type I diabetes mellitus, Inflammatory bowel disease, Antifolate resistance, Leishmaniasis, Legionellosis, and Asthma. The enriched local network cluster in the STRING database (Figure 2F) were JAK-STAT signaling pathway, and Interleukin-1 family, JAK-STAT signaling pathway, IL-6-type cytokine receptor ligand interactions, and Positive regulation of NK T cell proliferation, JAK-STAT signaling pathway, Mixed, incl. Type III interferon signaling pathway, and Interferon receptor activity, and IL-6-type cytokine receptor ligand interactions. The enriched reactome terms (Figure 2G) were Interleukin-10 signaling, Signaling by Interleukins, Interleukin-4 and Interleukin-13 signaling, Interleukin-12 signaling, Transcriptional regulation of white adipocyte differentiation, CD163 mediating an anti-inflammatory response, PPARA activates gene expression, Interleukin-6 family signaling, Leishmania infection, and Gene and protein expression by JAK-STAT signaling after Interleukin-12 stimulation. And the top 10 enriched Wiki-pathways (Figure 2H) were COVID-19 adverse outcome pathway, Cytokines and inflammatory response, altered glycosylation of MUC1 in tumor microenvironment, Ulcerative colitis signaling, IL-10 anti-inflammatory signaling pathway, Immune infiltration in pancreatic cancer, Development and heterogeneity of the ILC family, Prostaglandin signaling, LTF danger signal response pathway, and ncRNAs involved in STAT3 signaling in hepatocellular carcinoma.

**FIGURE 3:**
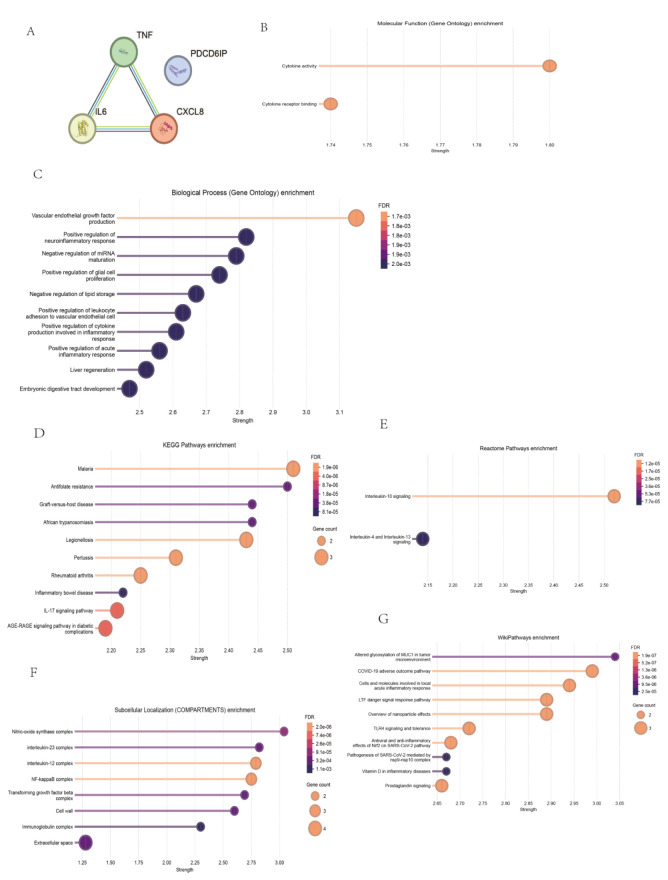
PPI and Enrichment analysis of Panel 2 **(A)** The PPI network of four inhibited genes in Panel 2. **(B)** The enriched GO BP terms based on genes in Panel 2. **(C)** The GO MF terms based on genes in Panel 2. **(D)** The enriched KEGG pathways based on genes in Panel 2. **(E)** The enriched reactome terms based on genes in Panel 2. **(F)** The enriched local network cluster in the STRING database based on genes in Panel 2. **(G)** The enriched Wiki-pathways based on genes in Panel 2.

For panel 2 (four inhibited genes), the PPI network was shown in Figure 3A. The enriched GO terms were presented in Figures 3B, 6C. Among GO enrichments, the top enriched BP terms include Vascular endothelial growth factor production, Positive regulation of neuroinflammatory response, Negative regulation of miRNA maturation, Positive regulation of glial cell proliferation, Negative regulation of lipid storage, Positive regulation of leukocyte adhesion to vascular endothelial cell, Positive regulation of cytokine production involved in inflammatory response, Positive regulation of acute inflammatory response, Liver regeneration, and Embryonic digestive tract development. There were no enriched CC terms. The two enriched MF terms were Cytokine activity and Cytokine receptor binding. The top enriched KEGG pathways (Figure 3D) were Malaria, Antifolate resistance, African trypanosomiasis, Graft-versus-host disease, Legionellosis, Pertussis, Rheumatoid arthritis, Inflammatory bowel disease, IL-17 signaling pathway, Viral protein interaction with cytokine and cytokine receptor, and AGE-RAGE signaling pathway in diabetic complications. The enriched reactome terms (Figure 3E) were Interleukin-10 signaling, and Interleukin-4/Interleukin-13 signaling. The enriched local network cluster in the STRING database (Figure 3F) were, Nitric-oxide synthase complex, interleukin-23 complex, interleukin-12 complex, NF-kappaB complex, Transforming growth factor beta complex, Cell wall, Immunoglobulin complex, and Extracellular space. And the top 10 enriched Wiki-pathways (Figure 3G) were Altered glycosylation of MUC1 in tumor microenvironment, COVID-19 adverse outcome pathway, Cells and molecules involved in local acute inflammatory response, Overview of nanoparticle effects, LTF danger signal response pathway, TLR4 signaling and tolerance, Antiviral and anti-inflammatory effects of Nrf2 on SARS-CoV-2 pathway, Vitamin D in inflammatory diseases, Pathogenesis of SARS-CoV-2 mediated by nsp9-nsp10 complex, and Prostaglandin signaling.

**FIGURE 4:**
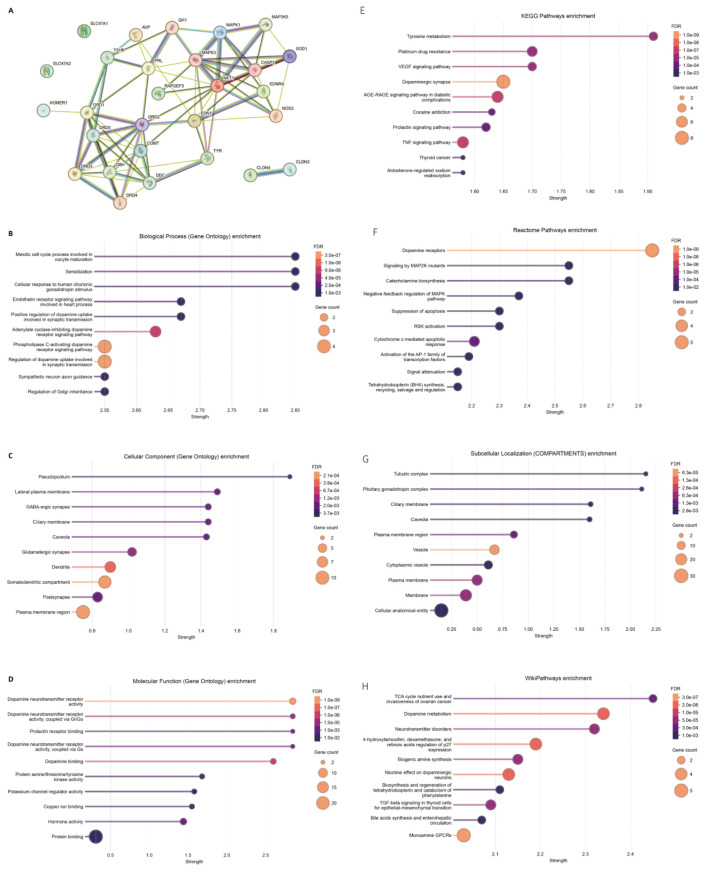
PPI and Enrichment analysis of Panel 3. **(A)** The PPI network of 28 MAGs in Panel 3. **(B)** The enriched GO BP terms based on genes in Panel 3. **(C)** The enriched GO CC terms based on genes in Panel 3. **(D)** The enriched GO MF terms based on genes in Panel 3. **(E)** The enriched KEGG pathways based on genes in Panel 3. **(F)** The enriched reactome terms based on genes in Panel 3. **(G)** The enriched subcellular localization terms based on genes in Panel 3. **(H)** The enriched Wiki-pathways based on genes in Panel 3.

For panel 3 (MAGs), the PPI network was shown in Figure 4A, and the hub genes include *AKT1, COMT, DBH, DDC, CASP3* and *DRD1*. Similarly, the enriched GO BP, CC and MF terms were presented in Figures 4B–D, respectively. The enriched KEGG pathways, reactome terms, subcellular localization terms, and Wiki-pathways were shown in Figures 4E–H, respectively.

### Differentially expressed genes in CRC among key-gene panels

**FIGURE 5:**
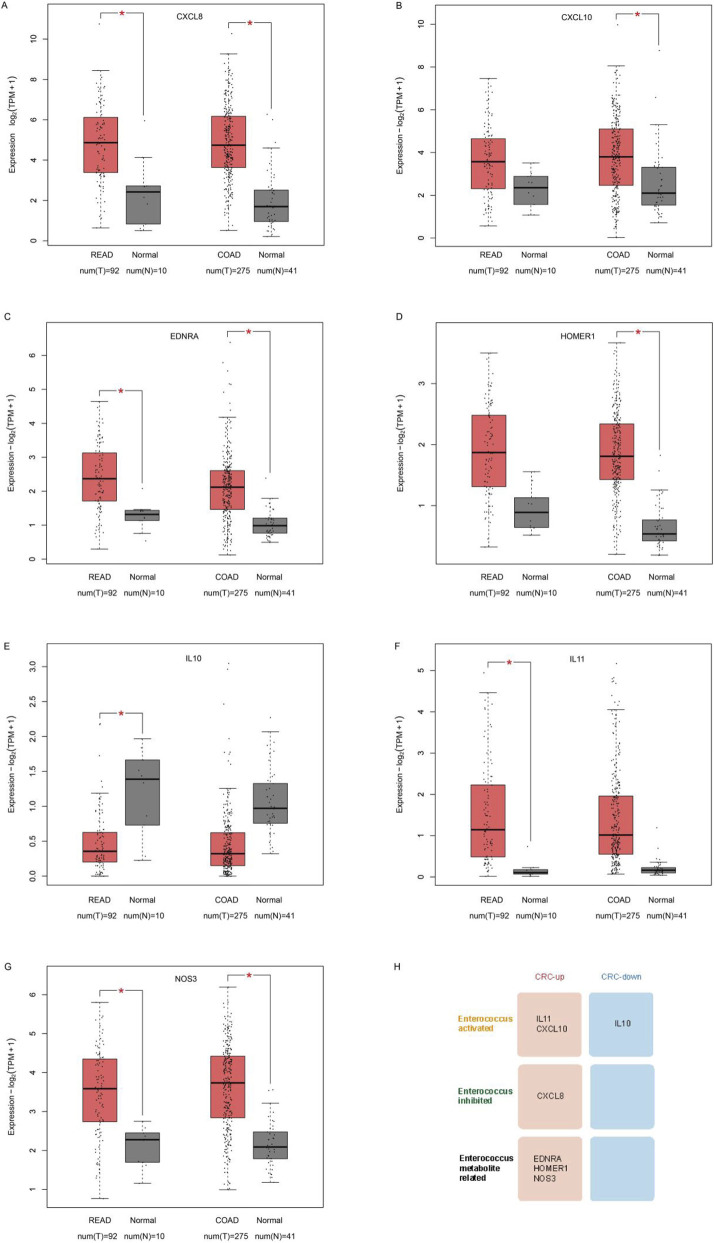
Expression changes of key genes in TCGA CRC samples **(A)** CXCL8 is upregulated in COAD and READ tumors. **(B)** CXCL10 is upregulated in COAD. **(C)** EDNRA is upregulated in both COAD and READ. **(D)** HOMER1 is upregulated in COAD. **(E)** IL10 is upregulated in READ. **(F)** IL11 is upregulated in READ. **(G)** NOS3 is upregulated in both COAD and READ. **(H)** Summarization of above results.

We analyzed differentially expressed genes in CRC among three panels (activated genes, inhibited genes, and MAGs). Among these key genes, *CXCL8* is upregulated in COAD and READ tumors (Figure 5A); *CXCL10* is upregulated in COAD (Figure 5B); *EDNRA* is upregulated in both COAD and READ (Figure 5C); *HOMER1* is upregulated in COAD (Figure 5D); *IL10* and *IL11* are upregulated in READ (Figures 5E,F); *NOS3* is upregulated in both COAD and READ (Figure 5G). The above results are summarized in Figure 5H. *IL11* and *CXCL10* are *enterococcus* activated genes upregulated in CRC; *IL10* is an *enterococcus* activated gene which is downregulated in CRC; *CXCL8* is an *enterococcus* inhibited gene upregulated in CRC; *EDNRA, HOMER1* and *NOS3* are MAGs upregulated in CRC.

### Links between CRC progression free survival and key genes

**FIGURE 6:**
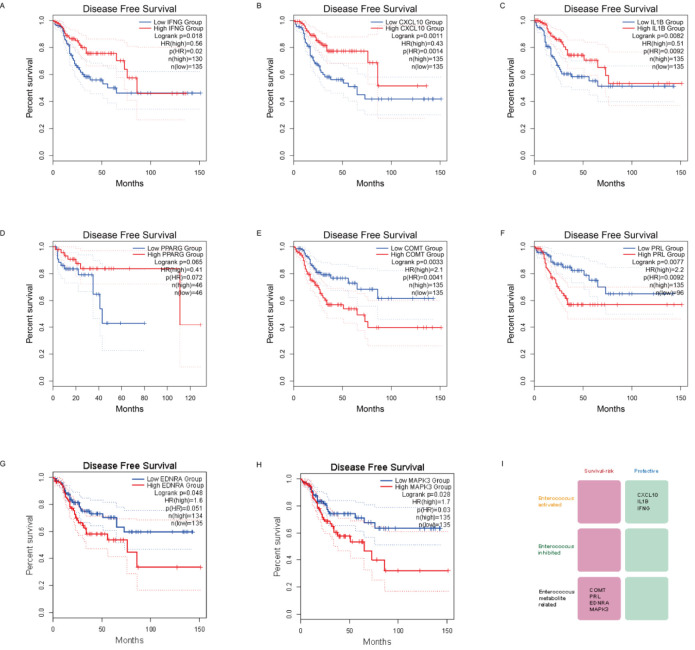
Links between CRC progression free survival and key genes. Among Panel one and 2, IFNG **(A)**, CXCL10 **(B)**, and IL1B **(C)** are protective genes in CRC survival (PFS). PPARG also plays a protective role, but it does not reach a significant level **(D)**. Among MAGs, there are four genes associated with CRC PFS, and they are all survival-risk genes: COMT **(E)**, PRL **(F)**, EDNRA **(G)**, and MAPK3 **(H,I)** Summarization of above results.

We probed links between CRC progression free survival and key genes. Among three panels of key genes, *IFNG* (Figure 6A), *CXCL10* (Figure 6B), and *IL1B* (Figure 6C) are protective genes in CRC survival (PFS). *PPARG* also plays a protective role, but it does not reach a significant level (P = 0.06, Figure 6D). Among MAGs, there are four genes associated with CRC PFS, and they are all survival-risk genes: *COMT* (Figure 6E), *PRL* (Figure 6F), *EDNRA* (Figure 6G), and *MAPK3* (Figure 6H), and the summary result is shown in Figure 6I.

### The influence of key genes on the tumor immune microenvironment

**FIGURE 7:**
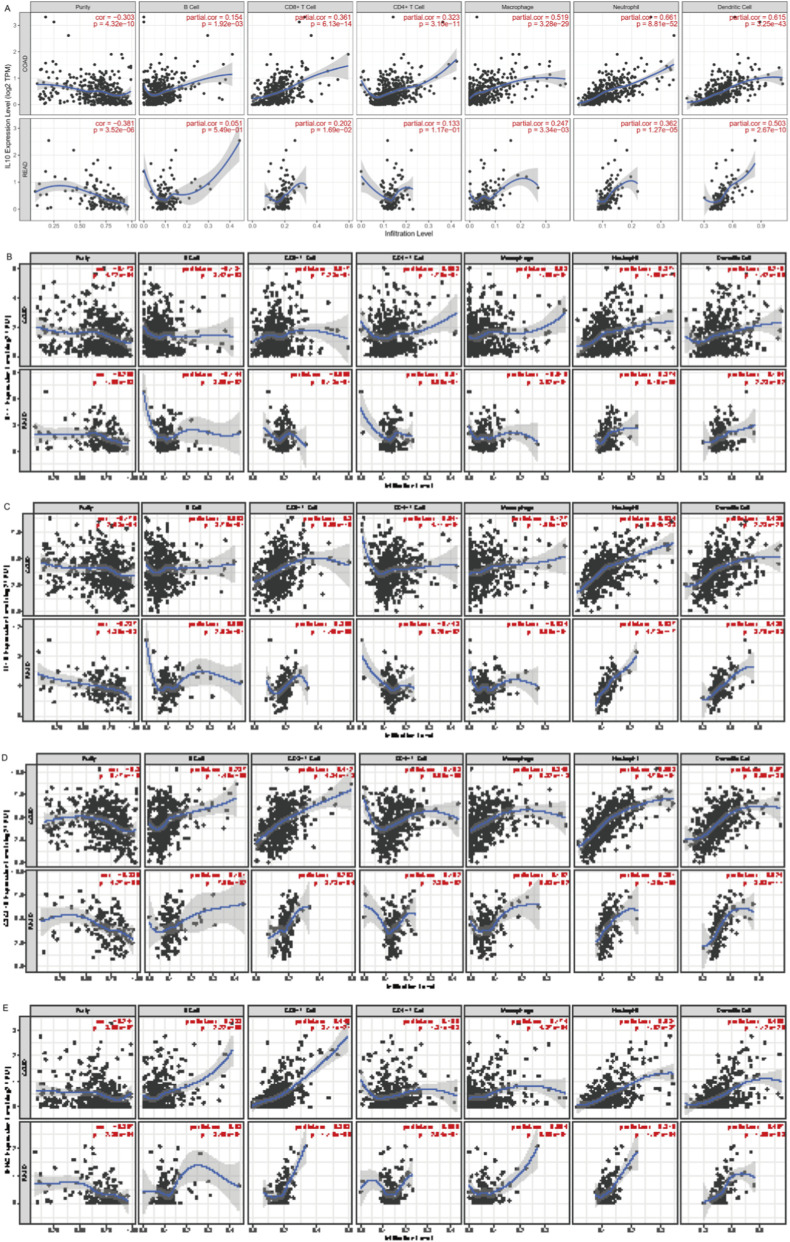
The influence of key genes in Panel one and two on the tumor immune microenvironment **(A)** Immune infiltration correlations between immune cells and IL10. **(B)** Immune infiltration correlations between immune cells and IL11. **(C)** Immune infiltration correlations between immune cells and IL1B. **(D)** Immune infiltration correlations between immune cells and CXCL10. **(E)** Immune infiltration correlations between immune cells and IFNG.

**FIGURE 8:**
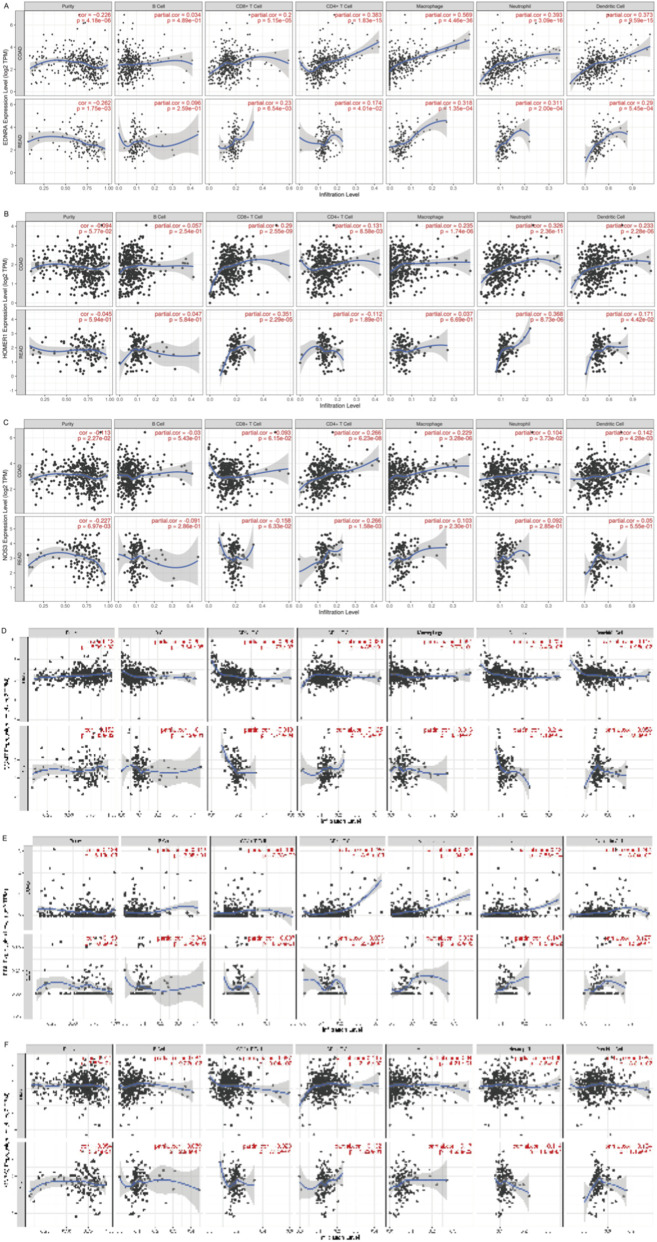
The influence of key MAGs on the tumor immune microenvironment **(A)** Immune infiltration correlations between immune cells and EDNRA. **(B)** Immune infiltration correlations between immune cells and HOMER1. **(C)** Immune infiltration correlations between immune cells and NOS3. **(D)** Immune infiltration correlations between immune cells and COMT. **(E)** Immune infiltration correlations between immune cells and MAPK3.

The correlation between immune cell levels and the key genes are shown in Figure 7. Among the *enterococcus* modulated genes, *IL10* is positively correlated with T cells, neutrophils, and dendritic cells (Figure 7A). Neutrophils and dendritic cells are positively correlated with *IL11* (Figure 7B), *IL1B* (Figure 7C), *CXCL10* (Figure 7D), and *IFNG* (Figure 7E). And CD8^+^ T cells are highly positively correlated with *CXCL10* and *IFNG*. Next, for MAGs (Figure 8), the trends of *enterococcus* modulated genes mentioned above are not obvious. Most correlations are around 0, especially, neutrophils are negatively correlated with *COMT* (Figure 8D) and *MAPK3* (Figure 8F).

### Experimental validation of key genes in CRC

**FIGURE 9:**
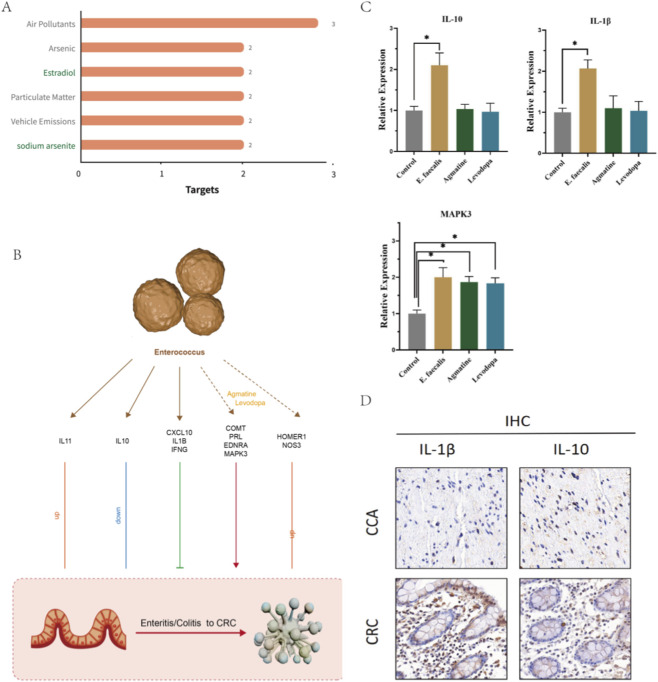
Candidate drugs for enterococcus-induced CRC development and the summary diagram of mechanism hypothesis of this study **(A)** the inhibitive chemicals towards the four risk genes, with at least two targets. Two drugs may be used to inhibit CRC development and are marked in green: estradiol and sodium arsenite. **(B)** The summary diagram of mechanism hypothesis of this study. **(C)** Validation of candidate gene expression in response to *Enterococcus faecalis* and its typical metabolites by qPCR. **(D)** Immunohistochemical (IHC) staining of IL-1βand IL-10 in clinical cancer tissue samples.

We performed a series of vitro experiments to validate the identified key genes mentioned before. We first assessed the mRNA expression of representative genes from our identified panels using qPCR in a cell model treated with *E. faecalis* and its typical metabolites—Agmatine and Levodopa. We found that *E. faecalis* treatment significantly upregulated the mRNA levels of *IL1B* and *IL10* compared to the control group. Interestingly, while the specific metabolites Agmatine and Levodopa did not significantly alter *IL10* or *IL1B* levels, both metabolites-along with *E. faecalis-*markedly induced the expression of *MAPK3* (Figure 9C). This result experimentally confirmed our prediction that *Enterococcus* modulates survival-risk MAGs through metabolic pathways, specifically highlighting the Agmatine/Levodopa-MAPK3 axis in CRC progression. Then we conducted immunohistochemical (IHC) staining on clinical CRC tissue samples (Figure 9D). The results demonstrated robust positive expression of *IL10* and *IL1B* characterized by distinct brown granular staining. Positive signals were primarily localized within the tumor stroma and infiltrating immune cells, supporting our immune infiltration analysis which suggested that these cytokines play active roles in the CRC immune microenvironment.

### Single-cell expression landscape of key genes in CRC

**FIGURE 10:**
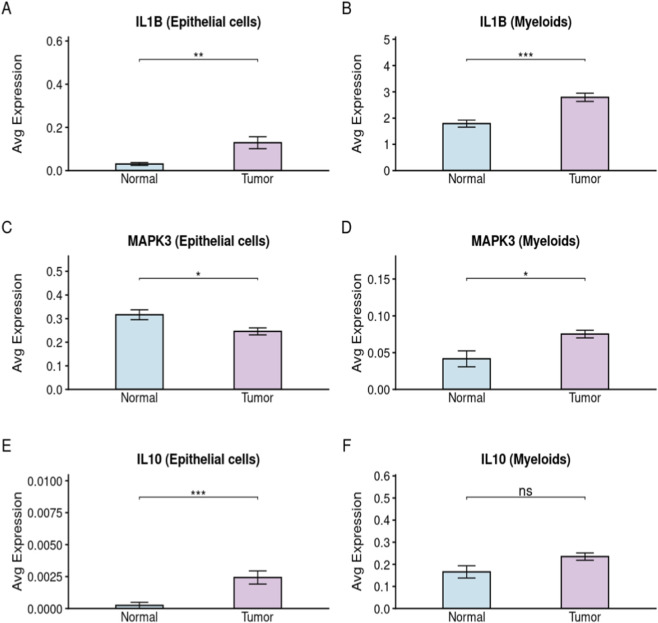
Single-Cell RNA Sequencing Reveals Cell-Type-Specific Expression Patterns of *IL1B, MAPK3,* and *IL10* in Colorectal Cancer Epithelial and Myeloid Cells **(A–F)** Bar plots showing the average expression levels of *IL1B, MAPK3,* and *IL10* in Epithelial cells (left) and Myeloid cells (right). Comparisons were made between Normal and Tumor tissue samples.

To gain high-resolution insights into the cellular origin and expression heterogeneity of the identified key genes, we analyzed the single-cell RNA sequencing dataset (GSE132465). The overall cell clustering, lineage annotation, and quality-control metrics confirming data reliability are presented in Figure S1. We specifically examined the expression of *IL1B, MAPK3,* and *IL10* in epithelial and myeloid cells, which are critical components of the intestinal barrier and immune microenvironment. *IL1B* exhibited a significantly higher expression level in both tumor epithelial cells and myeloid cells compared to normal tissues, indicating its pervasive pro-inflammatory role in the CRC microenvironment (Figures 10A,B). For the survival-risk gene *MAPK3*, single-cell analysis revealed that it was upregulated in tumor epithelial cells (Figure 10C), aligning with its function in promoting tumor cell proliferation. Interestingly, its expression in myeloid cells was also increased in tumor tissues (Figure 10D). Regarding *IL10*, we observed a upregulation in tumor epithelial cells (Figure 10E), whereas its expression in myeloid cells showed no significant difference (Figure 10F). These single-cell level findings further validate our bioinformatics and experimental results, suggesting that *Enterococcus* and its metabolites may modulate these genes in specific cell types to drive CRC progression.

### Candidate drugs for enterococcus-induced CRC development

To screen the inhibitive chemicals towards the four risk genes, and the drugs with at least two targets are listed in Figure 9A. Air pollutants, particulate matters, and vehicle emissions are not usable drugs. Pure arsenic is also a highly toxic substance and has significant intestinal toxicity, it should not be used to treat enteritis. Only two drugs may be used to inhibit CRC development and are marked in green: estradiol and sodium arsenite. In the future, it is worthwhile to explore the efficacy and molecular mechanisms of these two drugs as adjuvants in the prevention and treatment of CRC.

## Discussion

In this study, we identified a series of important metabolites (such as agmatine, formate, and levodopa) and human genes (such as *IL10, IL11, CXCL10, IL1B, IFNG, EDNRA, HOMER1, NOS3, MAPK3,* and *PRL*) that influence the development of CRC. We proposed three key gene panels and, based on them, put forward a series of molecular and immune mechanisms that influence CRC development, as well as some potential drugs for treating enterococcus-induced CRC.

In addition, we summarized the roles played by the above-mentioned key genes and drew a diagram of their mechanisms of action in CRC (Figure 9B). *Enterococcus* may activate the expression of *IL10, IL11, CXCL10, IL1B,* and *IFNG* in the human intestinal epithelial microenvironment, and alter the expression of *EDNRA, HOMER1, COMT, NOS3, MAPK3*, and *PRL* through typical metabolites such as agmatine and levodopa. Among these genes, the upregulation of *IL11, HOMER1,* and *NOS3* is positively correlated with CRC development, but whether they are oncogenes that drive CRC progression remains uncertain; *IL10* expression is negatively correlated with CRC development; *CXCL10, IL1B,* and *IFNG* may inhibit CRC progression; while *COMT, EDNRA, PRL,* and *MAPK3* may directly promote CRC development. In summary, the role of *enterococcus* in the progression from enteritis/colitis to CRC is complex, with a clear correlation, but it has both tumor-suppressing and tumor-promoting effects. This may depend on different *enterococcus* strains, as well as the combined effects of different metabolites and gene regulatory axes. For example, *enterococcus* faecium appears to benefit from its antimicrobial resistance, while *enterococcus faecalis* is recognized for its higher pathogenic potential (Archambaud et al., 2024).

The controversial role of *enterococcus* in CRC has been reported previously. Taking *enterococcus faecalis* as an example, the harmful role of *enterococcus faecalis* is associated with its ability to generate ROS and extracellular superoxide that can cause genomic instability, damaging colonic DNA, and because of that, predisposing the host to mutations and thus cancer (de Almeida et al., 2018; Fearon, 2011). Additionally, when primary colonic epithelial cells from mice were exposed to *enterococcus faecalis*, the Wnt/β-catenin signaling was activated, and pluripotent transcription factors associated with dedifferentiation were induced. This result demonstrated the role of *enterococcus faecalis* in inducing CRC (Wang et al., 2017). Meanwhile, *enterococcus faecalis* is a Firmicutes member, sometimes used as a probiotic product (Habermann et al., 2001; Gong et al., 2017). One of the first manifestation of CRC is spinal infection due to *enterococcus faecalis* (Manoku et al., 2023). In 2022, a study applied a murine CRC cell line and found that *enterococcus faecalis* can promote the migration and invasion of CRC cells, which dependents on its ability to activate pro-uPA, a key element of the urokinase-plasminogen system (Williamson et al., 2022). In 2023, Chinese scholars reported that *enterococcus faecalis* is a “driver bacteria” of CRC, which promotes the progression of CRC via its metabolite biliverdin through the PI3K/AKT/mTOR pathway (Zhang et al., 2023).

However, at present, there are still many gaps in comprehensive research on how the *Enterococcus* species affects the development of CRC, and our current study provides some new perspectives. First, we propose that the mechanism by which *enterococcus* influences the development of CRC involves two key metabolites: agmatine and levodopa. Research on the impact of these two metabolites on the development of CRC is still preliminary, with only some indirect evidence available. Limiting arginine-rich foods can suppress CRC, and agmatine is a product from arginine (Wei et al., 2023). Moreover, knockdown of agmatinase (which is an enzyme that hydrolyzes agmatine to putrescine and urea) can attenuate inflammation and tumorigenesis in a mouse model of colitis-associated CRC (Wang et al., 2023). In animal models, the levels of several agmatine was significantly increased in CRC rats vs. controls (Liu et al., 2018). Therefore, it is reasonable to hypothesize that agmatine is a metabolite that promotes CRC development. Our results showed that agmatine is a high-abundance metabolite of *enterococcus*, which may have a carcinogenic effect mainly through EDNRA (Figure 3B and 14B). Also, the effect of levodopa on CRC is currently poorly understood, and there are no direct studies on this topic. In breast cancer, levodopa appears to have a high degree of selectivity for tumor cells (Yosefi et al., 2025), and it has been used to improve the targeting of anticancer drugs. In addition, levodopa has shown some activity against pancreatic cancer in clinical studies, but the reliability of this effect is currently very weak (Yang et al., 2025). Besides, levodopa has some links to cancer anorexia (Herreros et al., 1999). In addition, levodopa can drive the secretion of prolactin (PRL), which stimulates prostate cancer growth (Lissoni et al., 2000). Our analysis also suggests that *enterococcus* may promote the development of CRC by driving PRL through levodopa. As Figures 3B, 9B shown, levodopa may exert its carcinogenic effects through three oncogenes: *COMT*, *PRL,* and *MAPK3*. Given that levodopa is clearly overexpressed in CRC (Figure 3A) and that its three targets have clear pro-CRC effects, following studies may preferentially target levodopa.

This study proposed three panels containing dozens of important genes, some of which played a core role. For example, among multiple oncogenes, *IL1B, CXCL10, IFNG, MAPK3, PRL,* and *COMT* were all important hub genes, meanwhile, they played survival-risk or protective roles. *IL1B* (gene IL-1β or IL1b) is an anti-CRC gene which confers benefits to PFS. In northern Caucasian cohort, IL1b C-3737T, G-1464C and PTGS2 T8473C variant genotypes are associated with risk of CRC compared to the homozygous wildtype genotype (Andersen et al., 2013). Moreover, as a pro-inflammatory cytokine, IL-1β diminished butyrate oxidation and NADH levels in CRC cells; and IL-1β suppresses basal mitochondrial respiration and lowered the mitochondrial spare capacity (Johnstone et al., 2017). In our results, *CXCL10* is another anti-CRC gene potentially impacted by *enterococcus*. TCGA data shows that elevated *CXCL10* expression in CRC tissues correlates with improved long-term survival and is inversely associated with lymph node infiltration and metastasis. Interestingly, a study published in 2024 revealed that a subset of cancer cells and macrophages are positive for *CXCL10* expression, and CXCL10-positive cells are predominantly located at the invasive front of the tumor, and there is an inverse correlation between serum *CXCL10* levels and its expression in cancer tissues (Li et al., 2024). This study implied that *CXCL10* may play a role in mediating the inflammatory responses at the invasive front in CRC and plays the distinct roles in CRC from serum (Li et al., 2024). *IFNG* is an important immune factor, and research consistently shows that it exerts an anti-cancer effect through immune activation (Ganapathi et al., 2014; Urbiola-Salvador et al., 2023; Yang et al., 2024). *MAPK3* has been widely proven to promote CRC by driving tumor cell proliferation through onco-signaling pathways such as AKT (Lin et al., 2023; Iskandar et al., 2024; Bhattacharya et al., 2016). There is currently limited evidence regarding the role of prolactin (PRL) in promoting CRC. But higher levels of *PRLR* expression were observed in the CRC cells and cell lines compared with normal colonic epithelial cells; and incubation of colon cancer cells with PRL-induced JAK2, STAT3 and ERK1/2 phosphorylation and increased expression of Jagged 1, which is a Notch-1 receptor ligand (Neradugomma et al., 2014). Therefore, PRL may enhance colon cancer stemness by modulating Notch signaling in a Jak2-STAT3/ERK manner. The catechol-O-methyltransferase (*COMT*) is another survival-risk gene in our results. However, there is currently controversy regarding its exact function and mechanism in relation to CRC. *COMT* has tumor-suppressive functions for CRC cell lines *in vitro* and *in vivo* experiments, where it inhibits CRC proliferation and invasion (Wu et al., 2015). *COMT* polymorphism is associated with CRC occurrence and malignancy (Wu et al., 2015; Jun et al., 2023; Hall et al., 2019). Theoretically, *COMT* may have anti-CRC effects by degrading levodopa, but our result shows that its high expression is associated with poorer PFS. Therefore, further cell and animal experiments are still needed to clarify the specific role of *COMT*. Finally, *EDNRA* is a clear oncogene in CRC. The *EDRNA* expression level markedly increases in CRC tissues, and patients with a high *EDNRA* expression exhibited significantly poor survival. In terms of mechanism, the EDN1/EDNRA/β-arrestin axis promotes CRC progression by regulating STAT3 phosphorylation (Lee et al., 2023). Hydroxytyrosol can exert an anti-CRC role by decreasing *EDNRA* expression through epigenetic modification (Del Saz-Lara et al., 2023). Together, research on *EDRNA*, *MAPK3*, *PRL*, and *COMT* remains in its infancy, and there is still significant room for further in-depth study.

At present, there are still some limitations in this study. First, through bioinformatics analysis, we have conducted an intensive analysis of the mechanism by which *enterococcus* influences the progression of inflammatory intestinal diseases to CRC. However, the current conclusions remain complex, and it is still too early to simply tell whether *enterococcus* plays an anti-cancer or pro-cancer role overall. Additionally, since the transition from chronic inflammation to cancer takes a long time, there is indeed controversy over whether some genes/metabolites promote or suppress CRC. A major reason is that the roles vary at different stages of CRC development. For example, IL-1β, as a pro-inflammatory cytokine, can activate the immune system and theoretically counteract the development of CRC, as our survival analysis has also confirmed. However, some studies suggest that IL-1β-related inflammatory signals may promote CRC invasion and migration (Chen et al., 2020). Therefore, future research should consider the factor of different time periods.

In summary, the role of *enterococcus* in the progression from enteritis/colitis to CRC is complex, with a clear correlation, but it has both tumor-suppressing and tumor-promoting effects. We identified three key gene panels and two typical metabolites that link this process. *Enterococcus* may activate the expression of *IL10, IL11, CXCL10, IL1B,* and *IFNG* in the human intestinal epithelial microenvironment, and alter the expression of *EDNRA, HOMER1, COMT, NOS3, MAPK3,* and *PRL* through typical metabolites such as agmatine and levodopa. Two drugs can be used as adjuvants in the prevention and treatment of CRC: estradiol and sodium arsenite.

## Data Availability

The original contributions presented in the study are included in the article/Supplementary Material, further inquiries can be directed to the corresponding author.
