# Supplementary material for: Enterococcus-driven metabolite-host gene networks in IBD-associated colorectal carcinogenesis: integrative multi-omics and experimental validation
Source: Front Cell Dev Biol. 2026 Mar 30;14:1793350. doi: 10.3389/fcell.2026.1793350 (PMC13071049; doi:10.3389/fcell.2026.1793350)
Supplement: Supplementary file 1 [file Supplementaryfile1.docx]

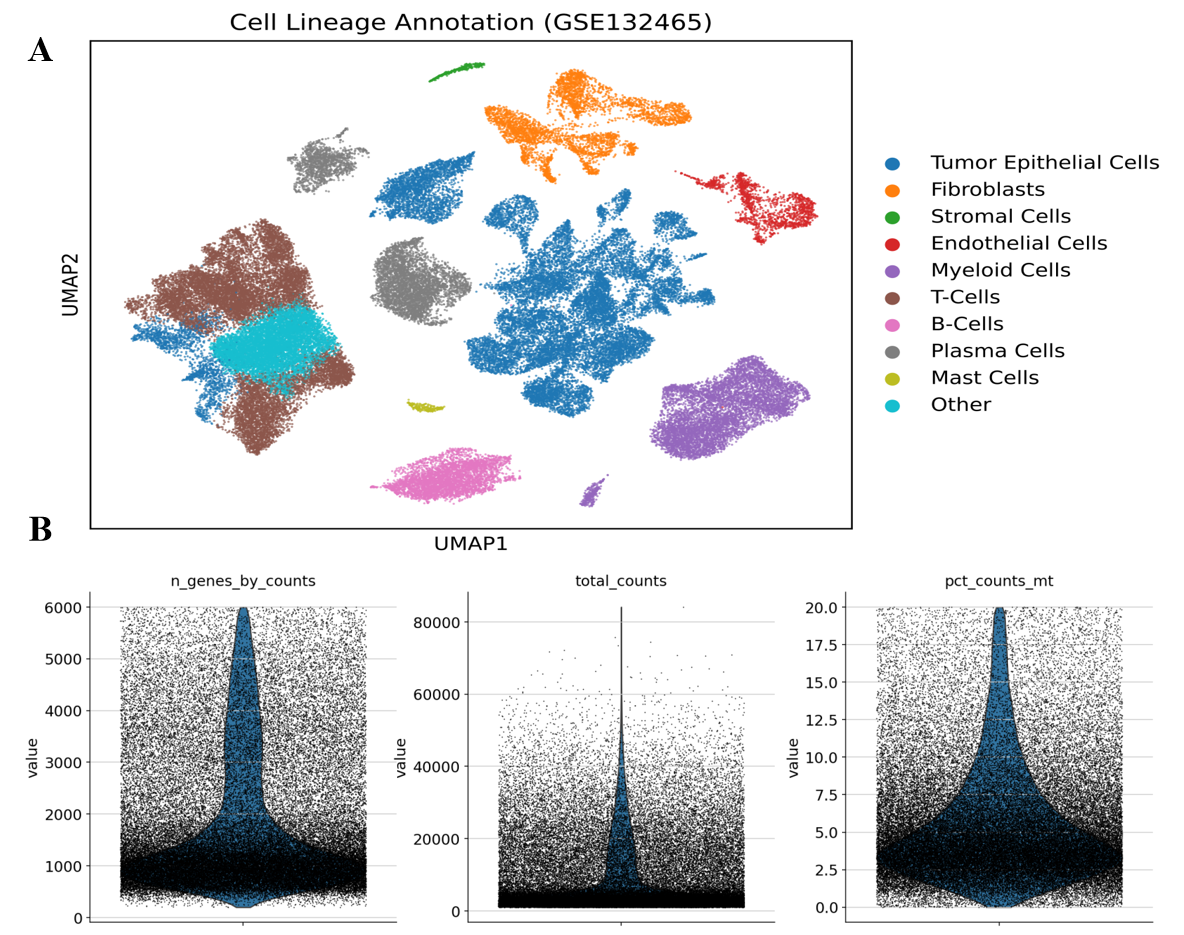


**Figure S1. Cell-type annotation and quality assessment of the GSE132465 scRNA-seq dataset.**

(A) UMAP visualization after dimensionality reduction showing cellular heterogeneity and annotation into ten major lineages based on canonical markers, including epithelial, stromal, endothelial, and immune populations.

(B) Violin plots with overlaid scatter points illustrating key quality-control metrics per cell—detected genes, total UMI counts, and mitochondrial gene percentage—demonstrating overall data quality and supporting subsequent filtering criteria.
